# Tuberculosis of the Lacrimal Gland: A Case Report

**DOI:** 10.7759/cureus.83871

**Published:** 2025-05-10

**Authors:** Anouar Ben Ameur El Youbi, Hajar El Youbi, Salma El Alaoui El Rhoul, Mohamed Afellah, Naouar Ouattassi, Mohammed Ridal, Najib Benmansour, Abdellatif Oudidi

**Affiliations:** 1 Otolaryngology - Head and Neck Surgery, Centre Hospitalier Universitaire Hassan II, Fes, MAR

**Keywords:** chronic epiphora, lacrimal gland, mycobacterium, orbital mass, tuberculosis

## Abstract

Tuberculosis of the lacrimal gland is a rare manifestation of extrapulmonary tuberculosis. It may occur in isolation or in association with pulmonary tuberculosis. The diagnosis is strongly supported by histopathological examination. Treatment relies on anti-tuberculous therapy. We report the case of a 53-year-old woman with no significant medical history who presented with chronic right-sided epiphora associated with the progressive appearance of a swelling in the region of the right lacrimal gland. A biopsy of the mass was performed, and histopathological analysis confirmed the diagnosis of tuberculous dacryoadenitis. The patient received anti-tuberculous therapy, which resulted in significant clinical improvement maintained over a one-year follow-up period. Primary mycobacterial infection of the lacrimal gland is extremely rare. Its clinical presentation is variable, making diagnosis challenging. Therefore, it should be considered in the differential diagnosis of any lacrimal gland hypertrophy, especially in endemic regions.

## Introduction

Tuberculosis is a chronic infectious disease caused by Mycobacterium tuberculosis (Koch bacillus), which has a high global disease burden, particularly in endemic regions. While it most commonly presents as a pulmonary infection following inhalation of aerosolized bacilli, it can also occur as an extrapulmonary disease via hematogenous dissemination [1]. Extrapulmonary tuberculosis often poses greater diagnostic challenges due to its atypical manifestations and the diversity of affected sites. Among these, lacrimal gland involvement is exceptionally rare and can mimic other, more common orbital pathologies, such as lymphoproliferative disorders or inflammatory pseudotumors. Biopsy and histopathological confirmation are essential for definitive diagnosis [2]. Initial management is primarily medical, based on anti-tuberculous antibiotic therapy [3].

In this case report, we present a rare instance of lacrimal gland tuberculosis. We describe the clinical presentation, radiological findings, histopathological features, and therapeutic management, and we discuss these aspects in light of existing literature.

## Case presentation

We report the case of a 53-year-old woman with no significant medical history and no known exposure to tuberculosis. She initially presented with chronic tearing (epiphora) of the right eye, which had been ongoing for approximately two months. Subsequently, she developed a progressively enlarging, painless swelling in the superolateral region of the right orbit, which had been evolving for about six months at the time of presentation. No systemic symptoms such as fever, night sweats, or weight loss were reported. On clinical examination, the swelling was firm, mobile, and non-tender, measuring approximately 3.5 cm in its greatest dimension. The overlying conjunctiva appeared normal, without redness or edema. There were no signs of local inflammation or infiltration into adjacent tissues. Visual acuity and intraocular pressure were within normal limits. The anterior segment and fundus examinations were unremarkable. Grade I ptosis was present, along with 3 mm of proptosis, compared to the contralateral side. No eyelid dystopia was observed. The remainder of the clinical examination was unremarkable.

Given the presentation, the differential diagnosis included chronic dacryoadenitis (including tuberculous or viral causes), benign epithelial tumors, and lymphoproliferative disorders.

**Figure 1:**
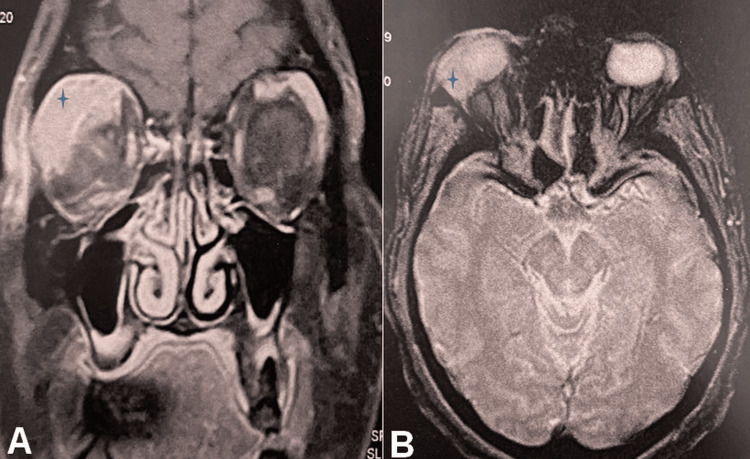
Coronal (A) and axial (B) MRI images showing a mass arising from the right lacrimal gland (star).

A cranio-orbital magnetic resonance imaging (MRI) was performed, revealing a 35 × 20 mm mass (height × width) centered on the lacrimal gland, showing intermediate signal intensity on T1- and T2-weighted images, high signal on diffusion-weighted imaging, low signal on ADC mapping, and enhancement after contrast administration, associated with mild grade I proptosis (Figure 1).

**Figure 2:**
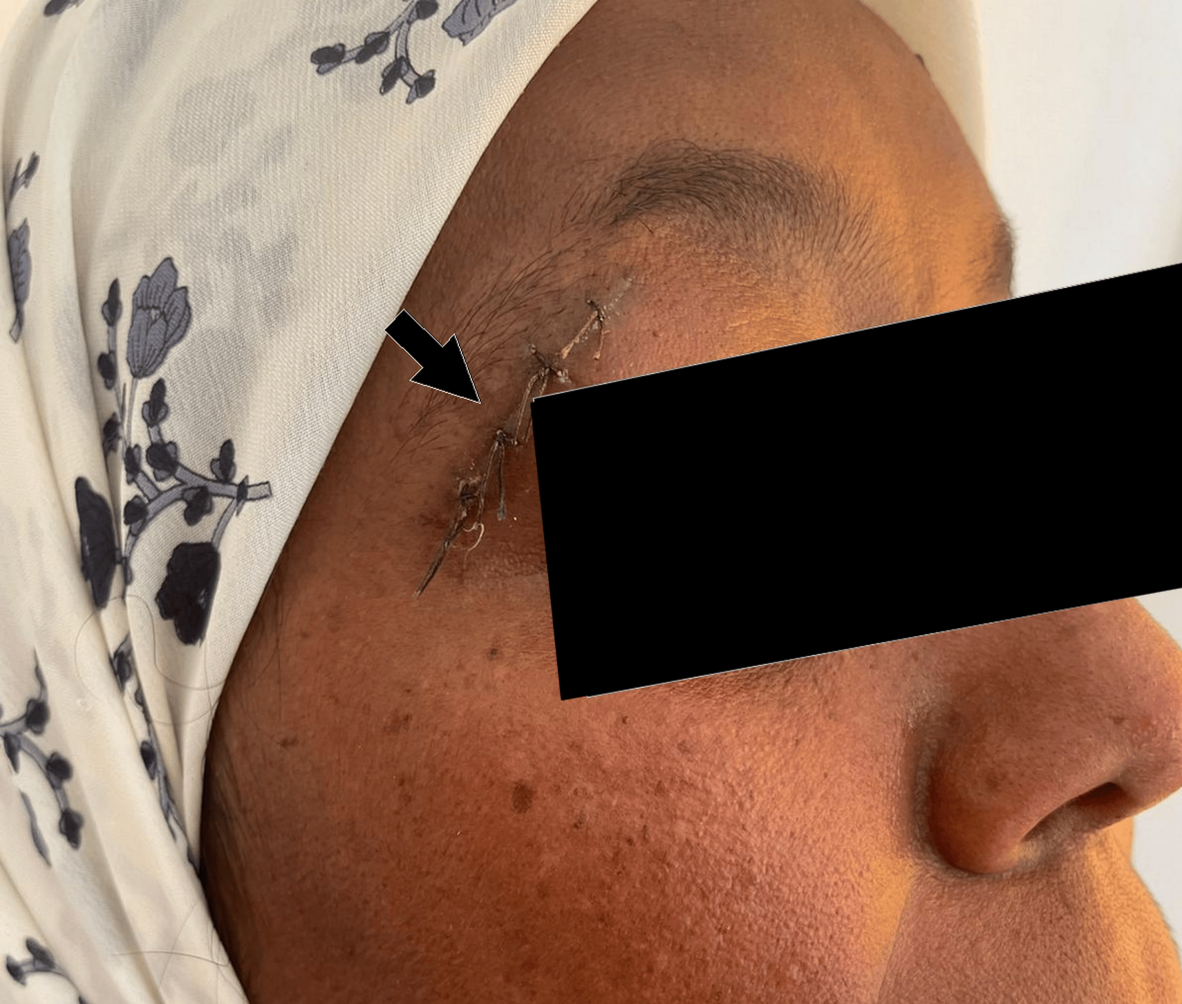
Clinical image of the biopsy site in the postoperative period (arrow).

A biopsy of the lacrimal gland mass was performed (Figure 2), and histopathological examination revealed granulomatous inflammation with caseating necrosis, consistent with lacrimal gland tuberculosis.

The patient was treated with anti-tuberculous antibiotics. The regimen included rifampicin, isoniazid, pyrazinamide, and ethambutol for the initial two months, followed by a six months continuation phase with rifampicin and isoniazid.

Clinical evolution was favorable, with regression of the swelling and no signs of recurrence observed after one year of follow-up.

## Discussion

Lacrimal gland tuberculosis is a rare condition, even in regions where tuberculosis is endemic [1]. It was first described by Abadie in 1881 [4]. The disease primarily spreads via hematogenous dissemination, with the lungs being the most common primary site [5]. However, it is essential to systematically search for a primary focus in all suspected cases of lacrimal gland tuberculosis [5]. In some cases, primary infection may occur through direct contact with contaminated hands or sputum containing bacilli [6]. Clinically, it may present as a superotemporal orbital swelling, usually painless and without overt inflammatory signs. It can also manifest as proptosis or regional lymphadenopathy [3].

The definitive diagnosis is based on the isolation of M. tuberculosis bacilli, requiring a tissue biopsy. The classic histopathological finding is an epithelioid granuloma with caseating necrosis [2]. Polymerase chain reaction (PCR) analysis of tissue samples for M. tuberculosis offers a rapid diagnostic alternative. Reported specificity is very high, ranging from 99.4% to 100%, while sensitivity ranges from 66.7% to 78%, surpassing that of acid-fast bacilli (AFB) staining (8.3%-47%) and culture methods (48%). However, the presence of PCR inhibitors, particularly in extrapulmonary specimens, may result in false-negative outcomes [7].

Radiologically, involvement of the lacrimal gland may appear as glandular enlargement or abscess formation. MRI, due to its multiplanar capabilities and lack of bone artifact, is particularly useful for assessing orbital masses. It helps differentiate among various types of lesions and can delineate the extent of involvement, including the lacrimal fossa and adjacent cerebral structures [8].

The differential diagnosis includes medial orbital subperiosteal abscess, orbital pseudotumor, lymphoma, and cavernous hemangioma. In pediatric populations, careful distinction must be made between orbital tuberculosis and other entities such as neuroblastoma, frequently associated with orbital bone erosion, and dacryoadenitis, an inflammatory condition of the lacrimal gland that may result from infectious etiologies, including syphilis, leprosy, cysticercosis, and schistosomiasis, or from systemic disorders such as lymphoma and sarcoidosis [9].

Initial treatment is medical. The initial treatment regimen for drug-susceptible extrapulmonary TB is similar to that for active pulmonary tuberculosis [3]. The therapeutic regimen typically includes rifampicin, isoniazid, pyrazinamide, and ethambutol during an eight-week intensive phase, followed by isoniazid and rifampicin for an additional 24-36 weeks [3].Surgical intervention is primarily indicated for obtaining histopathological confirmation [9]. In general, the prognosis is almost always favorable [10].

## Conclusions

Primary tuberculosis of the lacrimal gland is a rare and often under-recognized entity that may mimic more common orbital pathologies. Its diagnosis requires a high index of suspicion, especially in endemic areas. Histopathological examination remains essential for confirmation, and appropriate anti-tuberculous treatment typically leads to a favorable outcome.
